# *Ckip-1 3’UTR* alleviates prolonged sleep deprivation induced cardiac dysfunction by activating CaMKK2/AMPK/cTNI pathway

**DOI:** 10.1186/s43556-024-00186-y

**Published:** 2024-06-14

**Authors:** Beilei Dong, Rui Xue, Jianwei Li, Shukuan Ling, Wenjuan Xing, Zizhong Liu, Xinxin Yuan, Junjie Pan, Ruikai Du, Xinming Shen, Jingwen Zhang, Youzhi Zhang, Yingxian Li, Guohui Zhong

**Affiliations:** 1 Nanjing University of Chinese Medicine, Nanjing, 210023 China; 2 National Key Laboratory of Space Medicine, China Astronaut Research and Training Center, Beijing, 100094 China; 3 Beijing Institute of Pharmacology and Toxicology, State Key Laboratory of Toxicology and Medical Countermeasures, Beijing Key Laboratory of Neuropsychopharmacology, Beijing, 100850 China; 4 Oujiang Laboratory (Zhejiang Lab for Regenerative Medicine, Vision and Brain Health), Wenzhou, Zhejiang 325041 China

**Keywords:** Sleep deprivation, Cardiac function, *Ckip-1 3'UTR*, CaMKK2/AMPK/cTNI pathway

## Abstract

Sleep deprivation (SD) has emerged as a critical concern impacting human health, leading to significant damage to the cardiovascular system. However, the underlying mechanisms are still unclear, and the development of targeted drugs is lagging. Here, we used mice to explore the effects of prolonged SD on cardiac structure and function. Echocardiography analysis revealed that cardiac function was significantly decreased in mice after five weeks of SD. Real-time quantitative PCR (RT-q-PCR) and Masson staining analysis showed that cardiac remodeling marker gene *Anp* (atrial natriuretic peptide) and fibrosis were increased, Elisa assay of serum showed that the levels of creatine kinase (CK), creatine kinase-MB (CK-MB), ANP, brain natriuretic peptide (BNP) and cardiac troponin T (cTn-T) were increased after SD, suggesting that cardiac remodeling and injury occurred. Transcript sequencing analysis indicated that genes involved in the regulation of calcium signaling pathway, dilated cardiomyopathy, and cardiac muscle contraction were changed after SD. Accordingly, Western blotting analysis demonstrated that the cardiac-contraction associated CaMKK2/AMPK/cTNI pathway was inhibited. Since our preliminary research has confirmed the vital role of Casein Kinase-2 -Interacting Protein-1 (CKIP-1, also known as PLEKHO1) in cardiac remodeling regulation. Here, we found the levels of the 3’ untranslated region of *Ckip-1 (Ckip-1 3’UTR)* decreased, while the coding sequence of *Ckip-1 (Ckip-1 CDS)* remained unchanged after SD. Significantly, adenovirus-mediated overexpression of *Ckip-1 3’UTR* alleviated SD-induced cardiac dysfunction and remodeling by activating CaMKK2/AMPK/cTNI pathway, which proposed the therapeutic potential of *Ckip-1 3’UTR* in treating SD-induced heart disease.

**Supplementary Information:**

The online version contains supplementary material available at 10.1186/s43556-024-00186-y.

## Introduction

Roughly one-third of a person’s life is spent sleeping [1–3]. However, an increasing number of people are experiencing sleep-related problems. Whether it’s due to forced overtime [4], insomnia, or poor lifestyle habits, such issues can all be classified as a form of sleep SD [5]. SD arising from various causes, is widespread and frequently disregarded by physicians as a treatable health problem. SD has been strongly associated with serious conditions including diabetes, hypertension, insulin resistance, obesity, obstructive sleep apnea, anxiety and depression. These medical and psychiatric comorbidities heighten the risk of heart attack and stroke for individuals [6, 7]. A study conducted by Stanford University found that for every 5% decrease in deep sleep time, the risk of premature death increases by 13-17% [8].

SD has wide-ranging effects on the body, including memory loss, lack of energy, and even sudden death. A majority of cases of sudden death caused by SD are related to the cardiovascular system [9, 10]. This suggests that the cardiovascular system is particularly susceptible to the impacts of SD. Several studies have demonstrated that SD can influence heart function [11]. While most previous studies on the effects of SD on the heart have focused on short-term effects [12–14], few have demonstrated the impact of long-term SD on cardiac function in experimental animals, and the underlying mechanisms are even less clear. Research has shown that SD can impact cardiovascular health by causing endothelial dysfunction, metabolic disorders, changes in sympathetic nervous system activity, and immune damage [15]. While SD has been associated with an increased risk and progression of conditions like arrhythmia, myocardial infarction, and hypertension, the specific mechanisms through which it affects the cardiovascular system remain poorly understood. Therefore, there is a need to develop targeted interventions to mitigate its detrimental effects.

The structure of mRNA consists of two main parts, including a coding sequence region (CDS) and untranslated regions (UTRs) at the 5’ and 3’ ends [16, 17]. The CDS and 3’ UTR of mRNA were considered to form a complete structure by means of one-to-one correspondence, still, it was unexpected to find out that 3’ UTR can participate in the regulation of life activities independently of its CDS-encoded proteins [18, 19]. CKIP-1 (also known as PLEKHO1) is a protein contains a pleckstrin homology domain at the amino-terminal, a carboxyl-terminal region rich in leucine, and five putative PXXP motifs [20, 21]. CKIP-1 protein is involved in the regulation of cell homeostasis, including proliferation, differentiation, apoptosis and morphology [22, 23]. Our previous researches have shown that CKIP-1 protein plays an essential protective role in pathological cardiac remodeling by inhibiting the phosphorylation of histone deacetylase 4 (HDAC4) through protein phosphatase 2A (PP2A) [24]. Employing RNA sequencing, RNA fluorescent in situ hybridization and RT-q-PCR, we observed diverse expression and localization of the 3’ UTR and CDS of Ckip-1 mRNA in cardiomyocytes [19]. What’s more, the 3’ untranslated region of *Ckip-1 (Ckip-1 3’ UTR)* has the ability to independently inhibit pathological cardiac remodeling by activating the CaMKK2/AMPK signaling pathway [19]. However, the role of CKIP-1 and it’s 3’ UTR in SD induced cardiac dysfunction remains unknown.

Calcium/calmodulin-dependent kinase kinase 2 (CaMKK2), is a kinase strongly induced by increase in intracellular Ca^2+^ levels resulting from the opening of voltage-gated Ca^2+^ channels [18], acting as a direct upstream kinase of AMP-activated protein kinase (AMPK) [25, 26]. AMPK is an important energy sensor regulating cell metabolism that responds to various stresses [27]. Furthermore, AMPK is involved in numerous biological processes, including regulation of insulin sensitivity, autophagy and cardiomyocyte contraction [24, 28–31]. Recently, cardiac troponin I (cTNI) has been identified as a significant substrate of AMPK. In experiments where isolated cardiomyocytes were co-incubated with AMPK agonists, phosphorylation of the Ser150 site of cTNI was observed. This phosphorylation led to an increase in myocyte relaxation time and improved contraction capability [24]. Therefore, we hypothesized whether *Ckip-1 3’UTR* could potentially play a role in regulating myocardial dysfunction resulting from SD through the CaMKK2/AMPK/cTNI pathway.

In this study, we subjected mice to five weeks of SD to investigate the effect of SD on cardiac function using a device with a rotating bar. Our findings revealed that SD can result in cardiac dysfunction and a decrease in the CaMKK2/AMPK/cTNI signaling pathway. Additionally, there was a significant decrease in the mRNA content of *Ckip-1 3’-UTR* but not *Ckip-1 CDS*. To our excitement, adenovirus-mediated overexpression of *Ckip-1 3’UTR* alleviated SD-induced cardiac dysfunction and remodeling by activating CaMKK2/AMPK/cTNI pathway, which proposed the therapeutic potential of *Ckip-1 3’UTR* in treating SD-induced heart disease. The *Ckip-1 3’UTR* utilized in this investigation essentially functions as a noncoding RNA. Nucleic acid-based drugs present numerous advantages over conventional medications, including heightened stability, reduced resistance, enhanced specificity, and prolonged therapeutic efficacy. This research not only illuminates fresh perspectives on the mechanisms driving the impact of SD on cardiac function but also provides valuable insights into prospective treatment avenues.

## Results

### SD leads to cardiac remodeling and dysfunction in mice

**Fig. 1:**
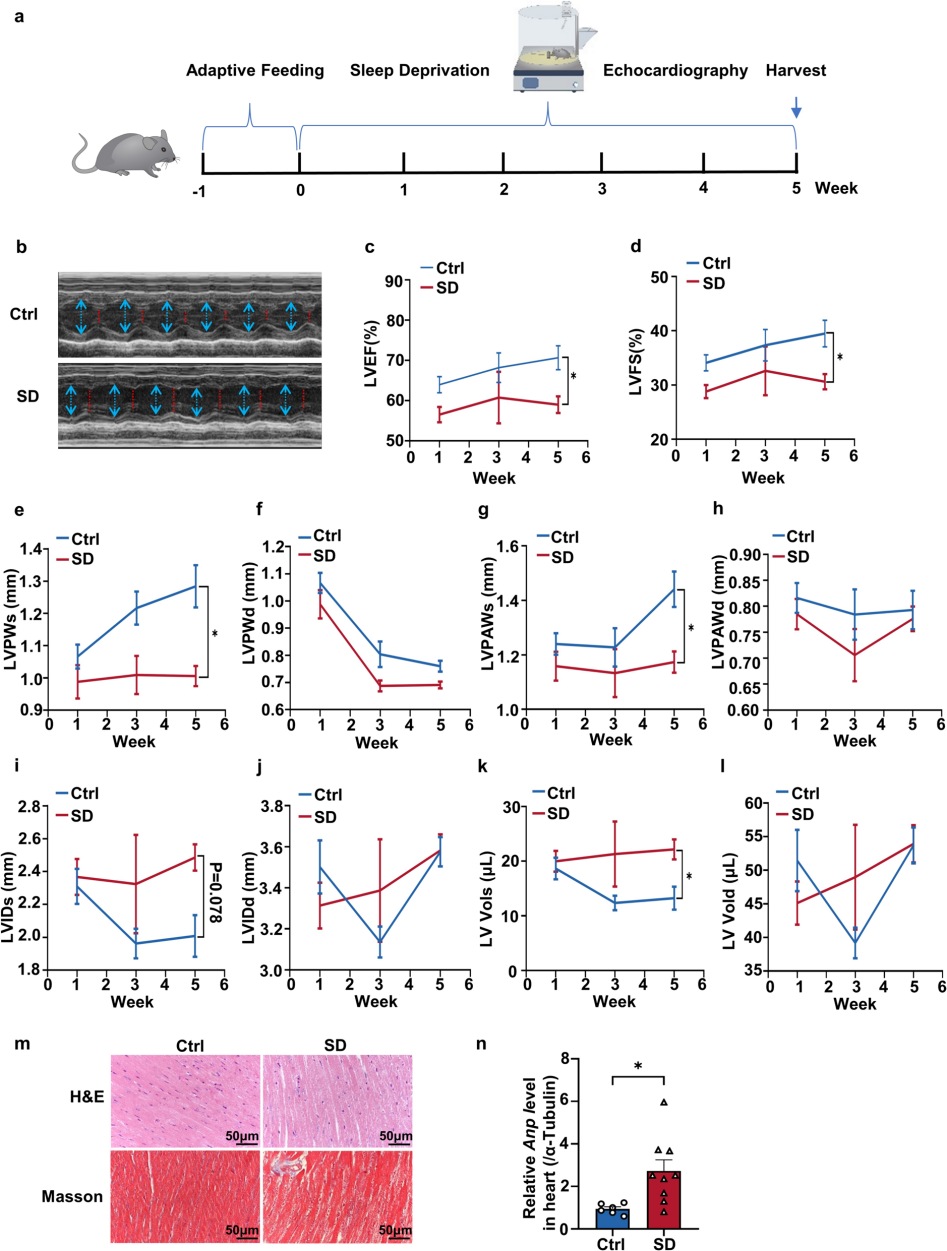
SD led to a decrease in the cardiac contractile function and changes in cardiac structure in mice. **a** Experimental model diagram. After a week of adaptive feeding, mice were subjected to SD for five weeks, echocardiography was conducted on the first, third and fifth week. **b** Representative M-mode echocardiographic images of Ctrl and SD groups after 5 weeks. **c** Left ventricular ejection fraction (LVEF) in Ctrl and SD mice. **d** Left ventricular fraction shortening (LVFS) in Ctrl and SD mice (Ctrl, *n *= 6; SD, *n* = 5). **e**-**h** The systolic and diastolic left ventricular posterior wall thickness (LVPWs and LVPWd, respectively) and systolic and diastolic left ventricular anterior wall thickness (LVAWs and LVAWd, respectively) by echocardiography. **i**-**j** The systolic and diastolic left ventricular internal diameter (LVIDs and LVIDd, respectively) in the indicated groups. **k**-**l** The end-systolic left ventricular volume (LV Vols) and the end-diastolic left ventricular volume (LV Vold) were measured by transthoracic echocardiography (Ctrl, *n* = 6; SD, *n* = 6). **m** Representative images of heart sections stained with Hematoxylin & eosin (H&E) and Masson trichrome staining. **n** cardiac remodeling marker gene Anp expression levels analyzed by quantitative real-time polymerase chain reaction (Ctrl, *n* = 6; SD, *n *= 9). Ctrl, control; SD, SD. Scale bar in sections, 50 μm. Anp, atrial natriuretic peptide. Data represent the means ± SEM. *p < 0.05; **p < 0.01

To investigate the impact of SD on cardiac function and structure, we exposed wild-type C57/B16J mice to a five-week SD using a device equipped with a rotating bar (Fig. 1a). Specifically, we turned off the device for one hour, four times a day, allowing the mice access to water and food. For the remaining 20 h, we turned the device on to disrupt their sleep. The control group received identical treatment except that they were not subjected to SD. Echocardiography was employed to evaluate the heart structure and function of the mice. From the echocardiography images, it was evident that the contractile function of the heart was impaired after SD (Fig. 1b). This was also reflected in alterations of left ventricular ejection fractions (LVEF) and left ventricular fraction shortening (LVFS), which are indicators of cardiac systolic function. It is evident from LVEF and LVFS that SD caused a decline in cardiac systolic function in the third week, showing a significant difference compared to the control group by the fifth week (Fig. 1c and d). To further investigate the effects of SD on left ventricular structure, we examined the cardiac structure by echocardiography in each experimental group of mice during the fifth week of SD. Following SD, the end-systolic left ventricular posterior walls thickness (LVPWs), end-systolic left ventricular anterior wall thickness (LVAWs) of mice were found to be significantly decreased in the heart (Fig. 1e and g). Additionally, the end-systolic Left Ventricular Volumes (LV Vols) were substantially larger in the hearts of sleep-deprived mice (Fig. 1k). SD had little effect on end-diastolic left ventricular posterior walls thickness (LVPWd), end-diastolic left ventricular anterior wall thickness (LVAWd), end-systolic and diastolic left ventricular internal diameter (LVIDs and LVIDd) and end-diastolic left ventricular volume (LV Vold) (Fig. 1f, h, i, j and l). Histological analysis was performed to evaluate the influence of SD on the heart. After SD, we observed gross evidence of edema characterized by separation of the myofibers in hematoxylin and eosin (H&E) staining sections, as well as a deeper staining of collagen in the Masson’s trichrome staining (MTT) sections (Fig. 1m). Furthermore, the expression level of fetal gene *Anp* was increased in the heart of mice after five weeks of SD (Fig. 1n). These results strongly suggest that SD can lead to a significant decrease in cardiac contractile function and cardiac remodeling.

### SD causes a reduction of *Ckip1-3’UTR* expression and inhibition of CaMKK2/AMPK/cTNI signaling pathway in the heart

**Fig. 2:**
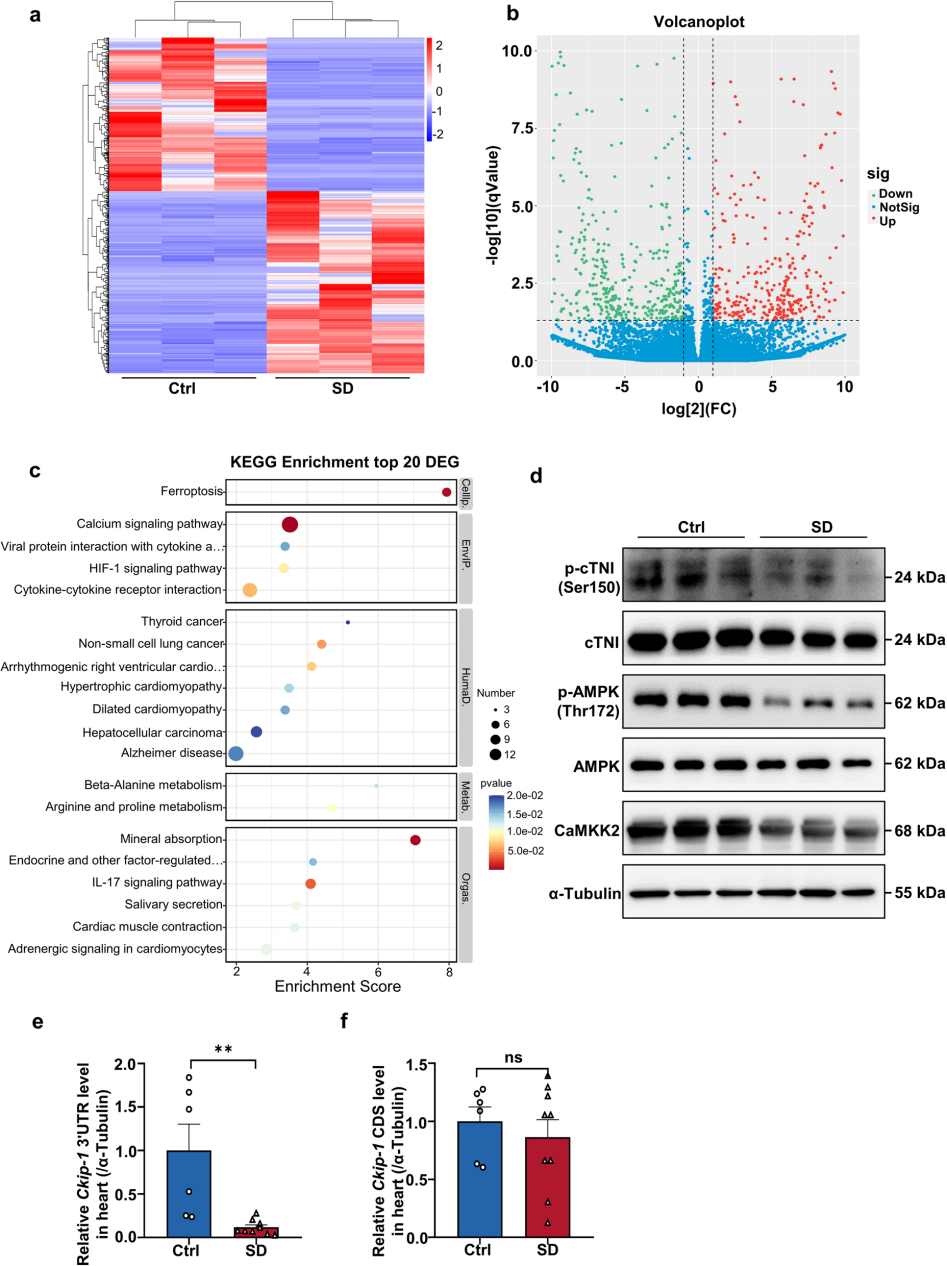
CaMKK2/AMPK /cTNI pathway changes in heart of mice after SD. **a** Heatmap of DEGs in SD and Ctrl mice heart using data generated by RNA sequencing that were analyzed with cut-off values of ± 2-fold change and *p*-value < 0.05, red represents high expression, blue represents low expression. **b** Volcano plot of DEGs, Red represents upregulated, and green represents downregulated, and other metabolites are labeled blue. **c** The KEGG enrichment analysis shows significantly altered pathways based on the top 20 DEGs. **d** Representative western blot analysis of p-cTNI, cTNI, p-AMPK, AMPK, and CaMKK2 proteins in cardiac tissue lysates from Ctrl and SD mice (these 3 samples are parallel samples). **e** Ckip-1 3 ′ UTR region mRNA expression level in heart extracts measured by quantitative real-time polymerase chain reaction. **f** Ckip-1 CDS region mRNA expression level in heart extracts measured by quantitative real-time polymerase chain reaction (Ctrl, *n* = 6; SD, *n* = 9). cTNI, cardiac troponin I; AMPK, AMP-activated protein kinase; CaMKK2, calcium/calmodulin-dependent kinase kinase 2; DEGs, differentially expressed genes; KEGG, Kyoto Encyclopedia of Genes and Genomes. Data represent the means ± SEM. **p < 0.01

To investigate the mechanism of SD on cardiac function, we conducted transcript sequencing analysis on mouse hearts. The heat map and volcano map indicated significant alterations the cardiac transcripts of mice in the SD group compared to the Ctrl group. Additionally, KEGG enrichment analysis of the top 20 DEGs revealed changes in genes associated with calcium signaling pathway, dilated cardiomyopathy, and cardiac muscle contraction following SD (Fig. 2a-c). Further, we examined several key signaling molecules associated with cardiac contractile function. As depicted in (Fig. 2d and Fig. S1a), the phosphorylation level of cTNI at Ser150 was found to be decreased in the hearts of mice subjected to 5 weeks of SD, consistent with the observed decline in cardiac contractile function. cTNI is one of the three subunits that constituting the troponin complex of the thin filaments in striated muscle. It has been reported that cTNI can be phosphorylated at Ser150 by AMPK [28], which in turn can modify the contractility of ventricular myocytes in mice. Interestingly, we also observed a decrease in the phosphorylation level of AMPK at Thr172 in the hearts of sleep-deprived mice. Our preliminary research has indicated that the 3’ untranslated region (3’UTR) of *Ckip-1* inhibits pathological cardiac hypertrophy through by activating of CaMKK2/AMPK signaling, independently of its cognate protein [20, 29]. In order to ascertain whether the regulation of *Ckip-1 3’UTR* and CaMKK2 is involved in the development of SD-induced cardiac dysfunction, we examined the expression of *Ckip-1 3’UTR* and the protein level of CaMKK2, and both were found to be decreased after SD while no difference in *Ckip-1* content was observed between the two groups (Fig. 2d and f and Fig. S1a). In summary, these findings suggest that SD leads to reduced expression of *Ckip-1 3’UTR* and inhibition of the CaMKK2/AMPK/cTNI signaling pathway in the heart. This raises the question of whether overexpression of *Ckip-1 3’UTR* could have beneficial therapeutic effects in SD-induced cardiac dysfunction.

### *Ckip-1 3′UTR* mitigates SD-induced cardiac dysfunction and structure change

**Fig. 3:**
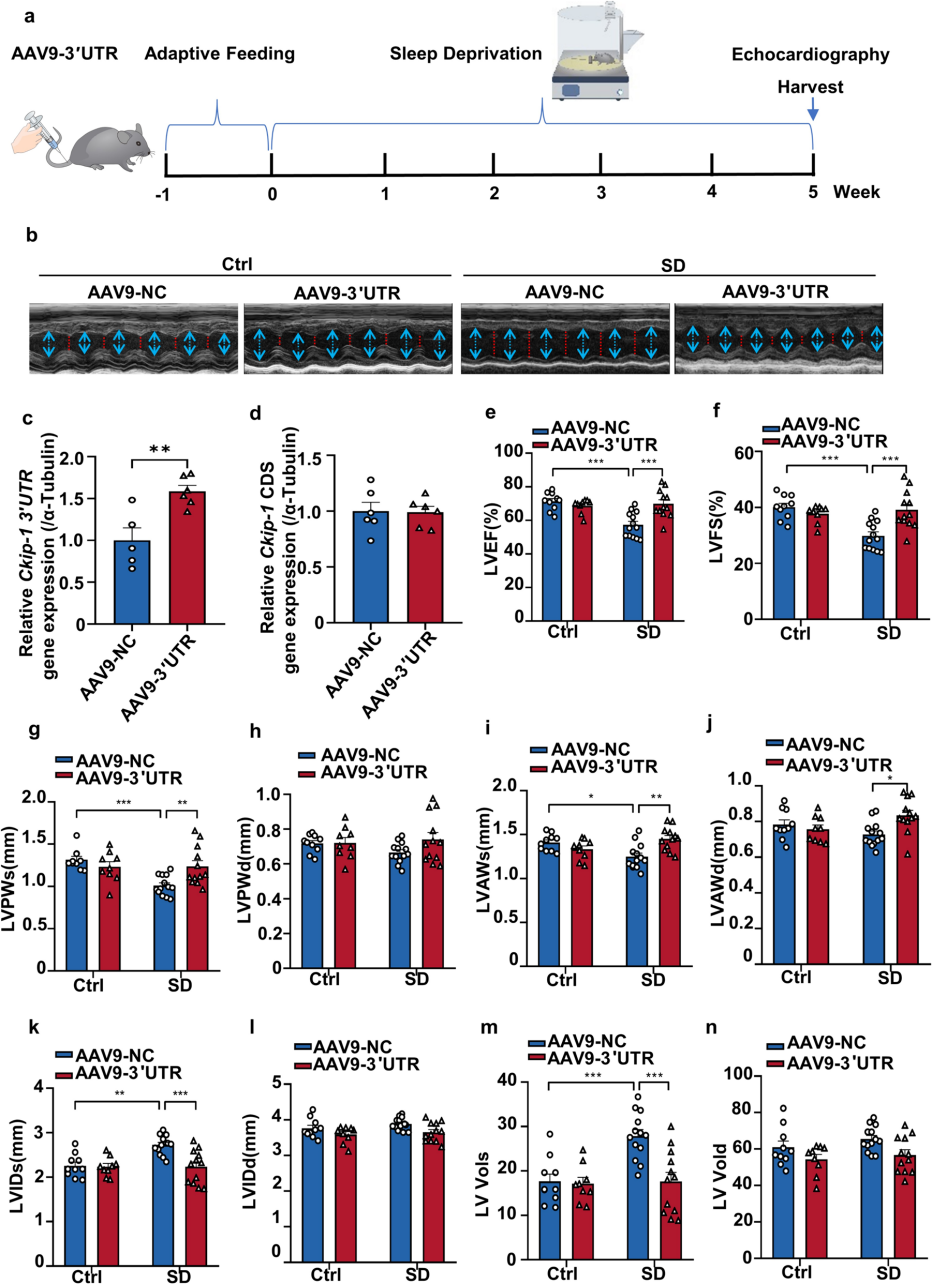
Administration of *Ckip-1 3’UTR* ameliorates SD-induced cardiac dysfunction and structure change. **a** After being injected with AAV9-*Ckip-1 3′ UTR* or AAV9-negative control (AAV9-NC) for a week, the wild-type mice were subjected to a five-week continuous SD modeling. **b** Representative M-mode echocardiographic images of each study group at 5 weeks. **c**-**d ***Ckip-1 3′ UTR* and CDS mRNA expression level in heart extracts measured by quantitative real-time polymerase chain reaction (AAV9-NC and AAV9-3′ untranslated region) (*n* = 6 in each group). **e** Left ventricular ejection fraction (LVEF). **f** Left ventricular fraction shortening (LVFS). **g**-**j** The systolic and diastolic left ventricular posterior wall thickness (LVPWs and LVPWd, respectively) and systolic and diastolic left ventricular anterior wall thickness (LVAWs and LVAWd, respectively) by echocardiography. **k**-**l** Quantitative analysis of the systolic and diastolic left ventricular internal diameter (LVIDs and LVIDd, respectively) in the indicated groups. **m**-**n ** The end-systolic left ventricular volume (LV Vols) and the end-diastolic left ventricular volume (LV Vold) were measured by transthoracic echocardiography (AAV9-NC-Ctrl, *n *= 10; AAV9-3′ UTR-Ctrl, *n* = 9; AAV9-NC-SD, *n* = 13; AAV9-3′ UTR-SD, *n* = 12). Data represent the means ± SEM. *p < 0.05, **p < 0.01, ***p < 0.001. NC, negative control; UTR, untranslated region

To further evaluate the therapeutic potential of *Ckip-1 3’UTR*, we utilized an adeno-associated virus (AAV) vector to introduce overexpression of *Ckip-1 3’UTR* under the control of the cardiomyocyte-specific cardiac troponin (cTnT) promoter. Mice were administered with 10^11^ AAV9-3’UTR viral particles one week prior to commencing the SD protocol. After five weeks of SD, we analyzed cardiac function (Fig. 3a). M-mode echocardiography reflected the excellent therapeutic effect of *Ckip-1 3’UTR* following SD (Fig. 3b). In the subsequent q-PCR experiment, we noted a notable elevation in the mRNA levels of *Ckip-1 3’UTR* in the 3’UTR-SD group compared to the NC-SD group, while no difference in *Ckip-1* content was observed between the two groups (Fig. 3c and d). Statistical analysis of LVEF and LVFS further supported the conclusion that injection of *Ckip-1 3’UTR* could restore heart contractile function in mice (Fig. 3e and f). Collectively, these results indicate that *Ckip-1 3’UTR* effectively prevents the decline in cardiac function induced by SD. Post SD, the administration of *Ckip-1 3’UTR* exhibited not only a protective effect on cardiac function but also induced structural changes. These alterations were reflected in various heart ultrasound indicators such as end-systolic left ventricular posterior wall thickness (LVPWs), end-systolic left ventricular anterior wall thickness (LVAWs), and the end-diastolic left ventricular anterior wall thickness (LVAWd) (Fig. 3g, i and j). The end-diastolic left ventricular posterior wall thickness (LVPWd), end-diastolic left ventricular internal diameters (LVIDd) and end-diastolic left ventricular volume (LV Vold) were not statistically significant differences (Fig. 3h and l, and 3n). Conversely, the end-systolic left ventricular internal diameters (LVIDs) and end-systolic Left Ventricular Volumes (LV Vols) were significantly higher in the NC-SD group compared to both the non-SD group and the 3’UTR-SD group (Fig. 3k and m). These results suggested that the overexpression of *Ckip-1 3’UTR* in heart mitigates SD-induced cardiac dysfunction.

### *Ckip-1 3’UTR *alleviates SD-induced cardiac remodeling and injury

**Fig. 4:**
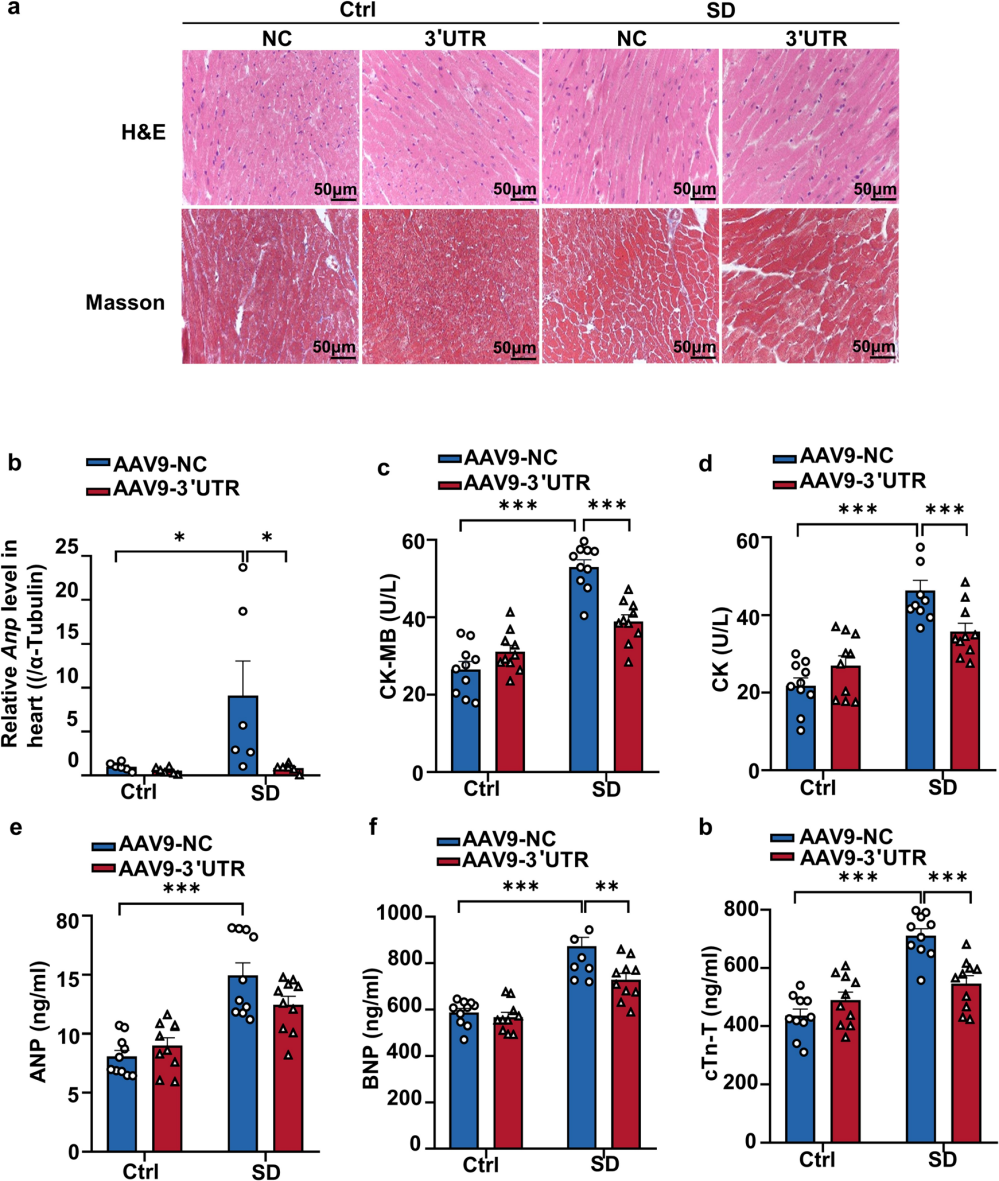
*Ckip-1 3′UTR* alleviates cardiac injury following SD. **a **Representative images of heart sections stained with Hematoxylin & eosin (H&E) and Masson trichrome staining. Scale bar in sections, 50 μm. **b** Cardiac remodeling marker genes *Anp* expression levels were assessed as indicated.**c-g** Results of CK-MB, CK, ANP, BNP, and cTn-T detected by serum myocardial zymogram (*n* =10 in each group). CK-MB, creatine kinase MB; CK, creatine kinase; ANP, atrial natriuretic peptide; BNP, brain natriuretic peptide; cTn-T, cardiac troponin T. Data represent the means ± SEM. *p < 0.05, **p < 0.01, ***p < 0.001

These findings illustrate the detrimental effects of SD modeling and the remarkable therapeutic potential of *Ckip-1 3’UTR*. H&E and Masson staining of heart tissues from the four groups further demonstrated that SD led to cardiac remodeling, while the injection of *Ckip-1 3’UTR* effectively alleviated this pathological alteration (Fig. 4a). The index of myocardial remodeling, *Anp*, showed a prominent increase in the NC-SD group, consistent with previous test results. However, the 3’UTR overexpression effectively countered this change (Fig. 4b). These results suggested that the overexpression of *Ckip-1 3’UTR* in cardiomyocytes alleviates SD-induced cardiac remodeling.

Detecting the myocardial enzyme spectrum is an effective method to confirm myocardial injury in clinical settings. When cardiomyocytes are damaged and rupture, intracellular myocardial enzymes are released into the bloodstream. Therefore, the level of serum myocardial enzymes is directly proportional to the extent of myocardial injury. We examined a range of indicators associated with cardiac injury, including CK-MB, CK, ANP, BNP, and cTn-T. The results revealed a significant increase in these indicators following SD, with myocardial injury being alleviated after the overexpression of *Ckip-1 3’UTR *(Fig. 4c and g). The findings further validated the cardiac damage caused by SD and the protective impact of *Ckip-1 3’UTR* on the heart.

### *Ckip-1 3’UTR* mitigates SD-induced cardiac remodeling by activating CaMKK2/AMPK/cTNI pathway

**Fig. 5:**
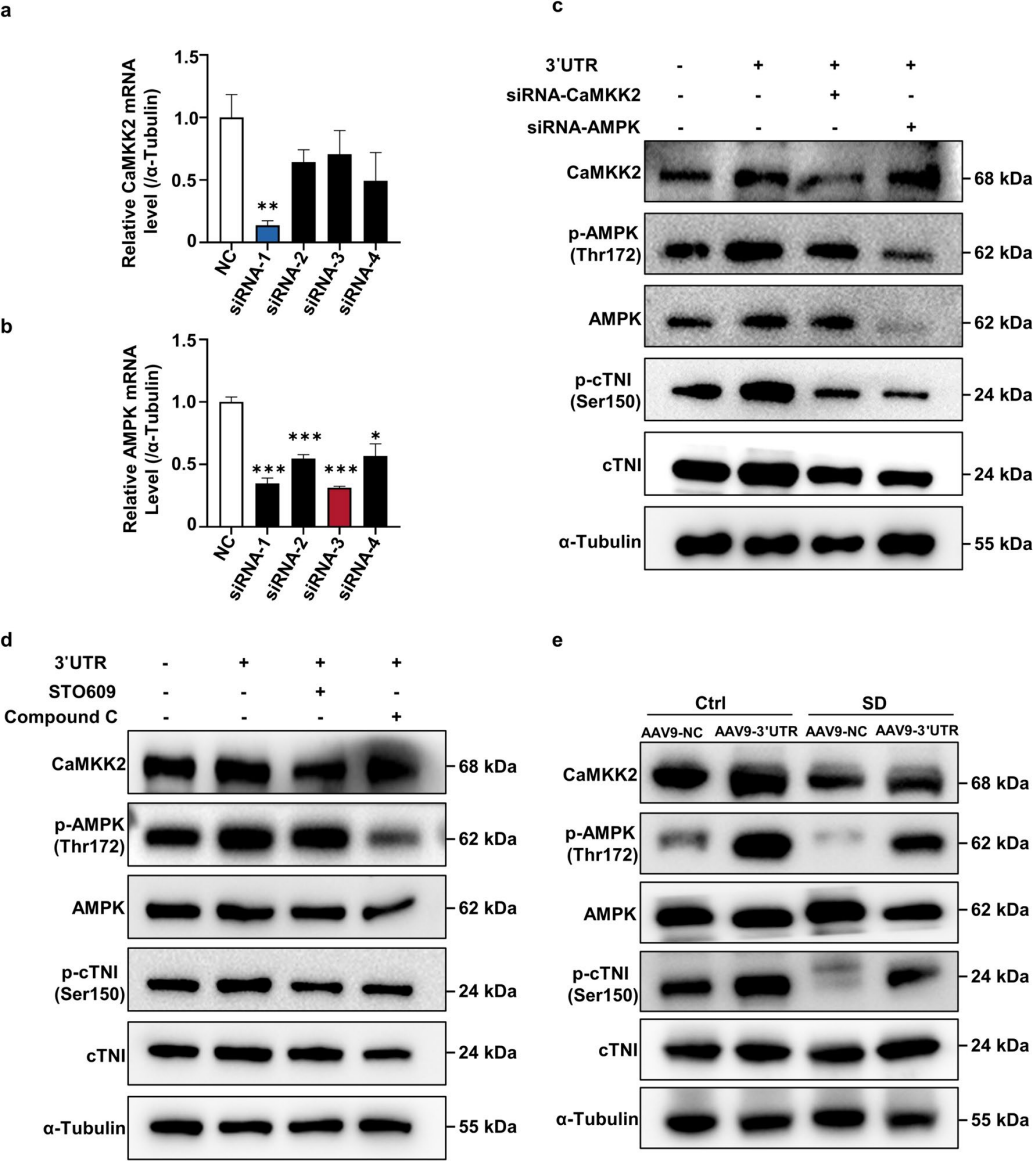
*Ckip-1 3′UTR* relieved SD-induced cardiac remodeling by activating CaMKK2/AMPK/TNI pathway. **a**-**b** The knockdown efficiency of CaMKK2 and AMPK siRNAs was assessed by Q-PCR, siRNA-1 of CaMKK2 (blue) and siRNA-3 of AMPK (red) were used in the following experiments. **c** CaMKK2, p-AMPK, AMPK, p-cTNI and cTNI protein expression in HL-1 cells transfected with 3′UTR, siRNA-CaMKK2, or siRNA-AMPK. **d** CaMKK2, p-AMPK, AMPK, p-cTNI and cTNI protein expression in HL-1 cells treated with 3′UTR, STO609 (inhibitor of CaMKK2, 10 μm), or Compound C (inhibitor of AMPK, 50 M), respectively, inhibitors were added 30 min before sample collection. **e** Western blotting images summarized the expression levels of CaMKK2, p-AMPK, AMPK, p-cTNI and cTNI in hearts from Ckip-1 3′ UTR and NC mice after SD. Data represent the means ± SEM. **p <  0.01, ***p <  0.001

**Fig. 6:**
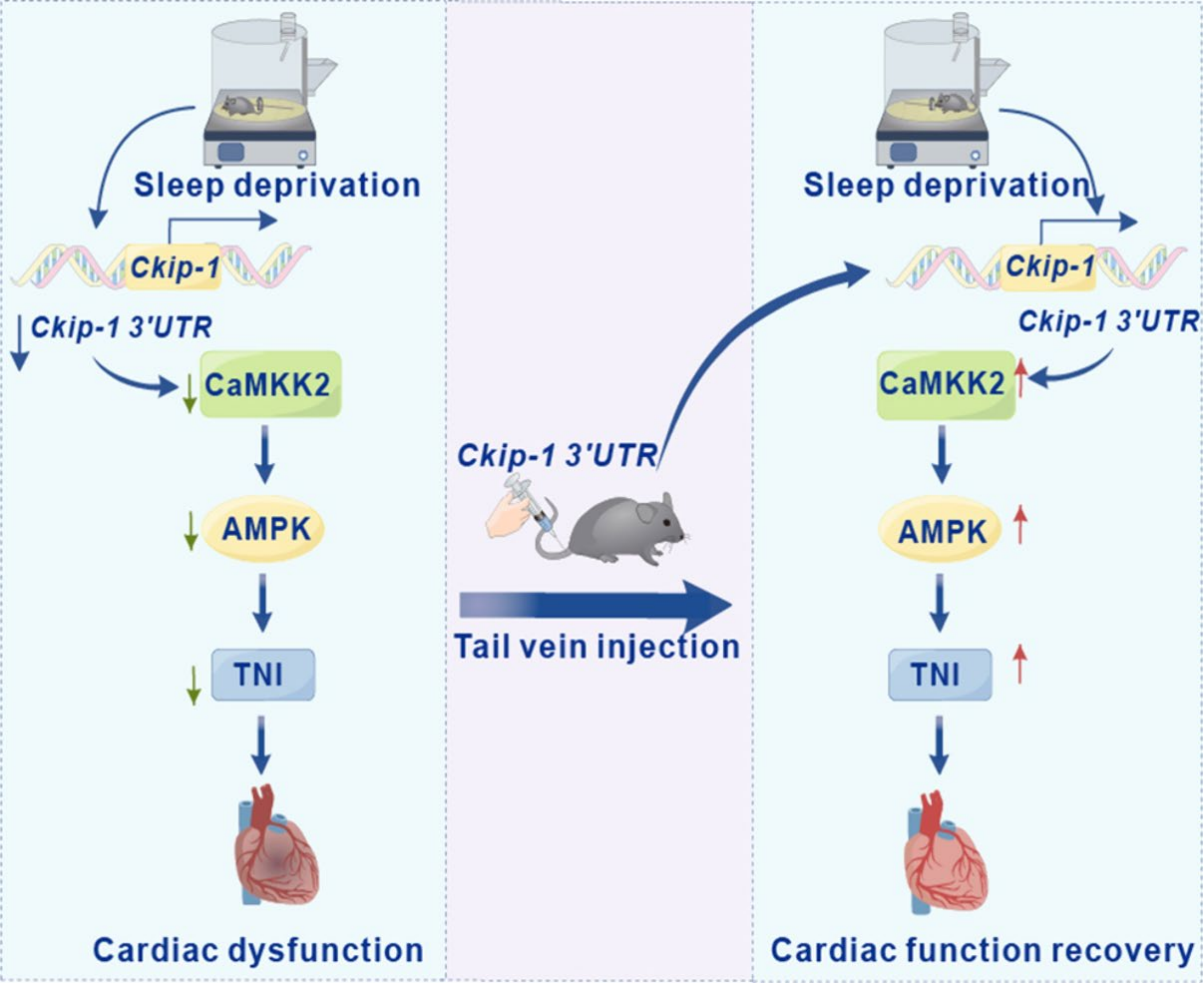
Schematic illustration of *Ckip-1 3′UTR* regulated cardiac function.  The model diagram of *Ckip-1 3′UTR* counteracting the decrease in cardiac function caused by SD through the CaMKK2-AMPK-TNI axis

 We found that SD caused cardiac dysfunction at the animal level with concomitant *CKIP-13’UTR* /CaMKK2/AMPK/cTNI pathway inhibition in the heart. However, whether the regulation of cTNI by *Ckip-1 3’UTR* is dependent on CaMKK2/AMPK was not elucidated. Therefore, we performed in vitro experiments in the mouse cardiomyocyte cell line HL-1 with siRNA and inhibitor of CaMKK2 and AMPK, respectively. The knockdown efficiency of CaMKK2 and AMPK siRNAs was assessed by Q-PCR, siRNA-1 of CaMKK2 (blue) and siRNA-3 of AMPK (red) had high knockdown efficiencies and were selected for subsequent experiments (Fig. 5a and b). In HL-1, overexpression of *Ckip-1 3’UTR* elevated cTNI phosphorylation, and that treatment using siRNA of both CaMKK2 and AMPK significantly counteracted the activation of cTNI by *Ckip-1 3’UTR *(Fig. 5c). Moreover, treatment with inhibitors of both CaMKK2 and AMPK significantly mitigated the activation of cTNI by *Ckip-1 3’UTR* as well (Fig. 5d). The above results suggest that the regulation of cTNI by *Ckip-1 3’UTR* is dependent on CaMKK2/AMPK. Finally, we examined the changes of CaMKK2/AMPK/cTNI pathway in heart of mice, and the results showed that the activity of this pathway was inhibited after SD in AAV9-NC mice, but activated in the AAV9-Ckip-1 3’UTR treated group (Fig. 5e and Fig. S2a-2c). Taken together, these findings suggest that overexpression of *Ckip-1 3’UTR* mitigates SD-induced cardiac remodeling and dysfunction through the CaMKK2-AMPK-cTNI axis (Fig. 6).

## Discussion

Our experiment sheds light on the impact of prolonged SD on the decrease of heart function and presents new protective measures. Prolonged SD in mice was found to be associated with a decline in cardiac systolic function and reduced CaMKK2/AMPK/cTNI pathway. SD caused an increase in the mRNA content of the myocardial remodeling marker *Anp*, while decreasing the mRNA level of *Ckip-1 3’UTR*. Tissue sections also showed signs of fibrosis disorder. Overexpression of *Ckip-1 3’UTR* in the heart effectively improved left ventricular ejection fraction (LVEF) and left ventricular fraction shortening (LVFS) to levels comparable to those of the non-SD group, even after a 5-week period of SD. Mechanically, *Ckip-1 3’UTR* overexpression promotes CaMKK2/ AMPK/TNI pathway. These findings highlight the impact of SD on cardiac function and demonstrate that *Ckip-1 3’UTR* can alleviate SD-induced cardiac dysfunction by activating the CaMKK2/AMPK/TNI pathway.

The importance of sleep in maintaining human health is widely recognized [32–35].The American Heart Association (AHA) recently updated their “8 Essentials for Life and Health,” including sleep duration as a new addition to the previously established “7 Rules for Simple Living” in 2010 [36–38]. Insufficient sleep has been linked to various diseases, with a significant association observed between SD and cardiovascular diseases [39–41]. Currently, it is understood that SD can impact cardiovascular function by affecting endothelial function [42, 43], immune function [15, 44–46], inflammation [47–49], sympathetic nervous system [50, 51], metabolism, and other factors [52, 53]. Studies have shown that key molecules and signaling pathways play important roles in the regulation of sleep homeostasis, e.g., the LKB1-SIK3-HDAC4 signaling axis in the brain is involved in sleep-wake transition regulation [54–56]. Research conducted by Xiamen University further indicated that chronic SD over a four-month period, using a modified “stick over water” method, could significantly impair cardiac function [57]. However, it remains unclear how exactly SD directly contributes to cardiac dysfunction [9]. In this study, we found that prolonged SD led to inhibition of CaMKK2/AMPK/cTNI signaling, resulting in decreased cardiac contractile function. This provides a basis for mechanistic elucidation and targeted treatment of the decline in cardiac contractile function due to SD.

AMPK plays a critical role in regulating sleep homeostasis, although it is not uniformly altered in different tissues following sleep homeostasis imbalance. SD for 6 h increased the phosphorylation level of AMPK and the mRNA level of CaMKK2 in the hypothalamus [58]. The sleep depth can be attenuated by the intracerebroventricular injection of AMPK inhibitor and enhanced by the intracerebroventricular injection of AMPK activator. What’s more, both inhibition and activation of AMPK modified the sleep response to SD, indicating that AMPK plays a central role in homeostatic sleep regulation [58]. However, the phosphorylation level of AMPK decreased in the hippocampus after chronic SD for 4 weeks in mice, ginsenoside Rg1 mitigated hippocampal mitochondrial dysfunction induced by chronic sleep deprivation and enhanced memory through the AMPK-SIRT3 pathway [59]. In our study, the activity of AMPK was inhibited in the heart of mice after 5 weeks of chronic SD, which was aligned with a previous study conducted by Song et al. [57]. The aforementioned studies indicate that AMPK plays a key role in regulating sleep homeostasis, but its change characteristics are inconsistent across organismal tissues. This suggests that AMPK is tissue-specific in the regulation of sleep homeostasis. In addition, differences in the degree and method of SD may also be an essential reason for the inconsistent changes in AMPK. Therefore, additional studies are warranted to systematically elucidate the role of AMPK in regulating sleep homeostasis.

There is currently no effective treatment available to protect the heart from the detrimental effects of SD. In recent years, nucleic acid drugs have garnered significant attention in research due to their potential to target a wide range of diseases, offer precise personalized therapy, ensure high safety, and provide long-lasting effects [60, 61]. Currently, there are three main directions for nucleic acid drug treatments. First, introducing normal genes to compensate for the functions lost due to gene mutations [62]. Second, inhibiting or silencing abnormal genes through RNA interference [63, 64]. And third, directly correcting abnormal gene structures through gene editing [65–70]. The *Ckip-1 3’UTR* used in this study is essentially a piece of noncoding RNA. Previous laboratory studies have demonstrated that *Ckip-1 3’UTR* can counteract pathological myocardial remodeling induced by pressure load change [19, 71]. Following SD, there is a significant reduction in the mRNA content of *Ckip-1 3’UTR*. The overexpression of AAV9 *Ckip-1 3’UTR* adenovirus can counteract the myocardial remodeling and decline in heart function induced by SD by activating the CaMKK2/AMPK/cTNI pathway. Previous studies have demonstrated that the *Ckip-1 3’UTR* can independently exert a protective effect on the heart, separate from its cognate protein [19, 71–74]. In our study, we also observed no significant change in *Ckip-1* mRNA expression after SD, further supporting the crucial role of the *Ckip-1 3’UTR* in cardiac protection during SD. Nucleic acid drugs offer several advantages over traditional drugs, including high stability, low resistance, high specificity, and long-lasting therapeutic effects [75, 76]. This study not only sheds new light on the mechanisms underlying the effects of SD on cardiac function but also offers insights into potential treatments.

In conclusion, our findings revealed that prolonged SD results in cardiac dysfunction and a decrease in the *Ckip-1 3’-UTR*/CaMKK2/AMPK/cTNI signaling pathway, overexpression of *Ckip-1 3’UTR* alleviated SD-induced cardiac remodeling and dysfunction by activating CaMKK2/AMPK/cTNI pathway, which proposed the therapeutic potential of *Ckip-1 3’UTR* in treating SD-induced heart disease.

## Materials and methods

### Animals

The mice used in this study were 2-month-old males with a C57BL/6J background, purchased from Biotechnology Co, Ltd. (Beijing, China) and raised in SPF animal center of China Astronaut Research and Training Center (CARTC), 12 h light/dark cycle, temperature controlled at 24 ± 1 °C. Animal experiments were performed in compliance with the guidelines for the use and care of live animals, and the experimental procedures were approved by the Animal Ethics Committee of CARTC (ACC-IACUC-2022-014).

### SD model

We conducted a five-week SD experiment in mice using a device with a rotary bar. The bar was set to rotate randomly and uninterruptedly when the mice entered sleep. We turned off the device for one hour, four times a day, allowing the mice access to water and food. For the remaining 20 h, we turned the device on to disrupt their sleep. The NSD group of mice were typically housed in cages but were given the same feeding schedule as the SD group.

### Transthoracic echocardiography

A high-resolution imaging system (Vevo 1100, Canada) was used for two-dimensional (2-D) guided M-mode echocardiography on mice anesthetized with tribromoethanol. We record 2-D images in parasternal long-axis and short-axis projections at the midventricular level. Each projection is measured in three beats to determine the size and thickness of the left ventricular cavity. Averaged LV wall thickness [anterior wall (AW) and posterior wall (PW) thickness] and internal dimensions at diastole and systole (LVIDd and LVIDs, respectively) are measured. LVFS is calculated using M-mode measurements [(LVIDd–LVIDs)/LVIDd ×100%]. LVEF was calculated from the 2-D short-axis view [( LV Vold-LV Vols)/LV Vold×100%].

### Histological analysis

The hearts of mice were collected after they were euthanized with cervical dislocation after five weeks of SD. The heart of the mice was fixed in 4% formalin (PH7.4) overnight. A paraffin embedding procedure was conducted after ethanol dehydration of the samples. Section (5 μm) were stained with Hematoxylin and Eosin (H&E) for routine histological examination. Masson’s trichrome staining was used to assess cardiac fibrosis.

### RT-q-PCR

Trizol (Invitrogen) was used to extract total RNA from mouse heart tissue or from HL-1 cells as instructed by the manufacturer. With the Superscript First-Strand Synthesis Kit (Takara), reverse transcription of RNA was performed to produce first-strand cDNA. The cDNA transcripts were measured using the SYBR Green (Takara) and Light Cycler (Eppendorf) systems. Results were normalized to α-Tubulin and reported as fold-change over control.

The following primers were used to analyze mRNA levels:

**Table:** 

Mouse-*Anp* forward:	5’-TTCGGGGGTAGGATTGACAG-3’,
Mouse-*Anp* reverse:	5’-CACACCACAAGGGCTTAGGA-3’;
Mouse-*Ckip-1 3' UTR *forward:	5’-GGGGGCAGGTCTGAAAT-3’,
Mouse-*Ckip-1 3' UTR* reverse:	5’-TGCAACATTTGGAGATAAAGAG-3’;
Mouse-*Ckip-1* CDS forward:	5’-CCGGATGGAAACCATCAGTCT-3’,
Mouse-*Ckip-1* CDS reverse:	5’-TCAGCACCACATAGCGGTTT-3’;
Mouse*-α-Tubulin*forward:	5’-GCAGATGCCCAGTGACAAGA-3’,
Mouse*-α-Tubulin* reverse:	5’-GTGCGCACCTCATCAATGACA-3’.

### RNA sequencing

RNA sequencing was conducted on hearts obtained from control (Ctrl) and SD mice. The Trizol reagent was used to extract total RNA as directed by the manufacturer. The NanoDrop 2000 spectrophotometer (Thermo Scientific, USA) was used to measure RNA purity and concentration, and the Agilent 2100 Bioanalyzer (Agilent Technologies, USA) to measure RNA integrity. The TruSeq Stranded mRNA LT Sample Prep Kit (Illumina, San Diego, CA, USA) was then used to prepare libraries. Differentially expressed genes (DEGs) analysis was performed using the R package DESeq2 (v 1.6.3), with a significance threshold set at *P* < 0.05 and a fold change threshold of < 0.5 or > 2. KEGG pathway enrichment analysis of DEGs between SD and Ctrl groups were performed using R package.

### Plasmids, siRNAs, inhibitors, and adenovirus

*Ckip-1 3′UTR* was obtained using standard PCR techniques from HL-1 and subsequently inserted into mammalian expression pGL3 vector (Promega). Shanghai GenePharma Co., Ltd. (Shanghai, China) synthesized the siRNAs. Their sequences are as follows:

**Table:** 

Mouse CaMKK2 siRNA-1	sense 5’-GCAAGAUCUUCUCCGGAAATT-3’,
	antisense 5’-UUUCCGGAGAAGAUCUUGCTT-3’;
Mouse CaMKK2 siRNA-2	sense 5’-GCAAGUGUACCAGGAGAUUTT-3’,
	antisense 5’-AAUCUCCUGGUACACUUGCTT-3’;
Mouse CaMKK2 siRNA-3	sense 5’-CCGAUAUAGCCGAAGACUUTT-3’,
	antisense 5’-AAGUCUUCGGCUAUAUCGGTT-3’;
Mouse CaMKK2 siRNA-4	sense 5’-CCUGGUAAAGACCAUGAUUTT-3’,
	antisense 5’-AAUCAUGGUCUUUACCAGGTT-3’.
Mouse AMPK siRNA-1	sense 5’-GCGUGUACGAAGGAAGAAUTT-3’,
	antisense 5’-AUUCUUCCUUCGUACACGCTT-3’;
Mouse AMPK siRNA-2	sense 5’-GGGAACACGAGUGGUUUAATT-3’,
	antisense 5’-UUAAACCACUCGUGUUCCCTT-3’;
Mouse AMPK siRNA-3	sense 5’-GCCUCACCCUGAAAGAGUATT-3’,
	antisense 5’-UACUCUUUCAGGGUGAGGCTT-3’;
Mouse AMPK siRNA-4	sense 5’-GCCUCACCCUGAAAGAGUATT-3’,
	antisense 5’-UACUCUUUCAGGGUGAGGCTT-3’.

Inhibitors of CaMKK2 (STO609, 10µM; Cayman) and AMPK (Compound C, CC,50 μm; Sigma) were added into HL-1 cells for 30 min, then proteins were extracted for WB analysis.

Adenovirus was conducted by HanBio Biotechnology (Shanghai, China). For Ckip-1 3’ UTR overexpression, the sequence of the mouse Ckip-1 3’ UTR was subcloned into the pHBAAV9-cTNT-MCS vector. AAV9-NC or AAV9-Ckip-1 3’ UTR were administered via the tail vein at a dosage of 10^11 viral particles per mouse, one week before SD. Subsequently, heart samples were collected at 5 weeks of SD for histological and functional analyses.

### Western blot

Heart tissues or HL-1 cell were lysed in lysis buffer with protease inhibitor cocktail on ice for 15–20 min. Polyvinylidene fluoride (PVDF) membranes were used to transfer the lysate after SDS-PAGE. After blocking with TBS supplemented with 5% (w/v) non-fat dry milk, the membranes were incubated with primary antibodies overnight. The antibodies used in the study are as follow: AMPK (CST; No.2532 S), p-AMPK(T172) (CST; No.2535 S), CaMKK2 (ABclonal; No. A9899), p-cTNI (Abcam; No.ab 169,867), cTNI (Proteintech; No. 21652-1-AP), and α-tubulin (Sigma; No.T9026). Horseradish peroxidase-conjugated anti-mouse and anti-rabbit secondary antibodies (ZSGB-Bio; cat. ZB-2305 and ZB-2301) were used to amplify signal of primary antibodies. Western blots were developed using an ECL chemiluminescent substrate (Millipore). ImageJ software (NIH, USA) was used for band intensity analysis.

### Myocardial zymogram detection

#### CK-MB/CK

Prepare 10 µl of blank sample, standard sample and sample to be tested respectively as required in the kit. Then add 200 µl of reagent one, mix the mixture evenly, and incubate at 37 ℃ for 5 min. Then add reagent two, mix well and start to measure after 3 min. Continuously measure for 2 min. Calculate the change value of absorbance (ΔA/min).

#### ANP/BNP/cTn-I/ cTn-T

1. After allowing the required flat noodles to reach room temperature equilibrium for 20 min, remove them from the aluminum foil bag. Seal the remaining flat noodles in a self-sealing bag and store them back at 4 °C; 2. Set up standard and sample wells, adding different concentrations of standard to each standard well in µL.; 3. Add 50 samples to the sample well for testing µ L; Blank holes are not added; 4. Except for blank wells, add detection antibodies labeled with horseradish peroxidase (HRP) to each well of the standard and sample wells one hundred µ L. Seal the reaction pores with a sealing film and incubate in a 37 ℃ water bath or constant temperature box for 60 min; 5. Discard the liquid, pat dry on absorbent paper, and fill each hole with detergent (350 µ L) .Let it stand for 1 min, shake off the washing solution, and use absorbent paper. Dry up and repeat washing the board 5 times (you can also use a washing machine to wash the board); 6. Add 50 substrates A and B each to each well µ L. Incubate at 37 ℃ the dark for 15 min; 7. In each well, add 50µ L stop solution within 15 min. Measure the OD values at 450 nm.

### Statistical analysis

All quantitative data in this study were presented as the mean ± SEM. A two-tailed unpaired Student’s t-test and two-way analysis of variance (ANOVA) were used for the analyzing of differences between experimental groups when appropriate. Statistical analysis was performed using Prism software (GraphPad prism, version 9.0) and differences were considered significant when *p < 0.05, ** *p* < 0.01, *** *p* < 0.001.

### Supplementary Information

Supplementary Material 1.

Supplementary Material 2.

## Data Availability

The datasets used and/or analyzed during the current study are available from the corresponding author on reasonable request.
