# Supplementary figures and images for: Ckip-1 3’UTR alleviates prolonged sleep deprivation induced cardiac dysfunction by activating CaMKK2/AMPK/cTNI pathway
Source: Mol Biomed. 2024 Jun 14;5:23. doi: 10.1186/s43556-024-00186-y (PMC11176284; doi:10.1186/s43556-024-00186-y)

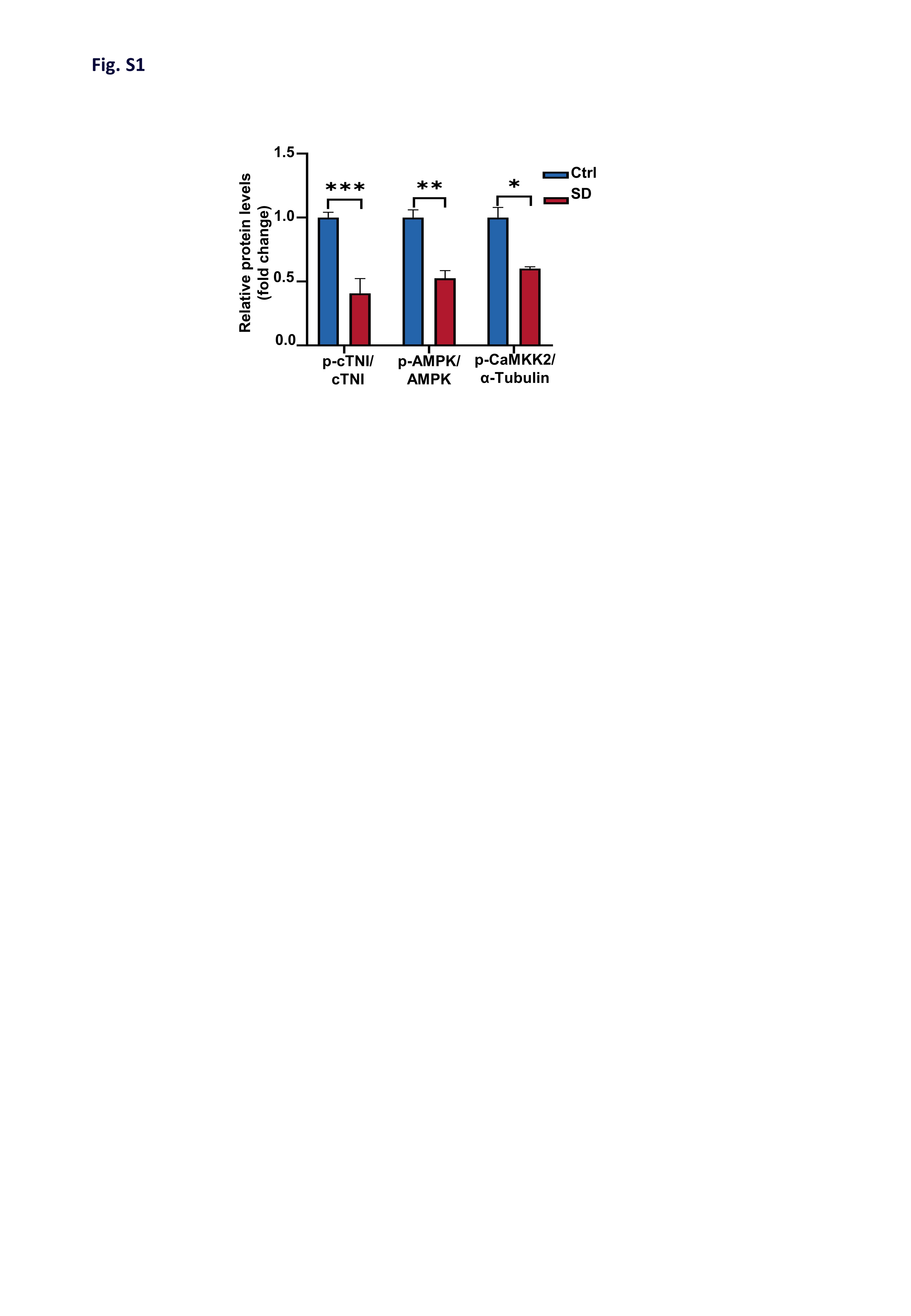

Supplement: Supplementary file 1 — Supplementary Material 1. [file 43556_2024_186_MOESM1_ESM.jpg]

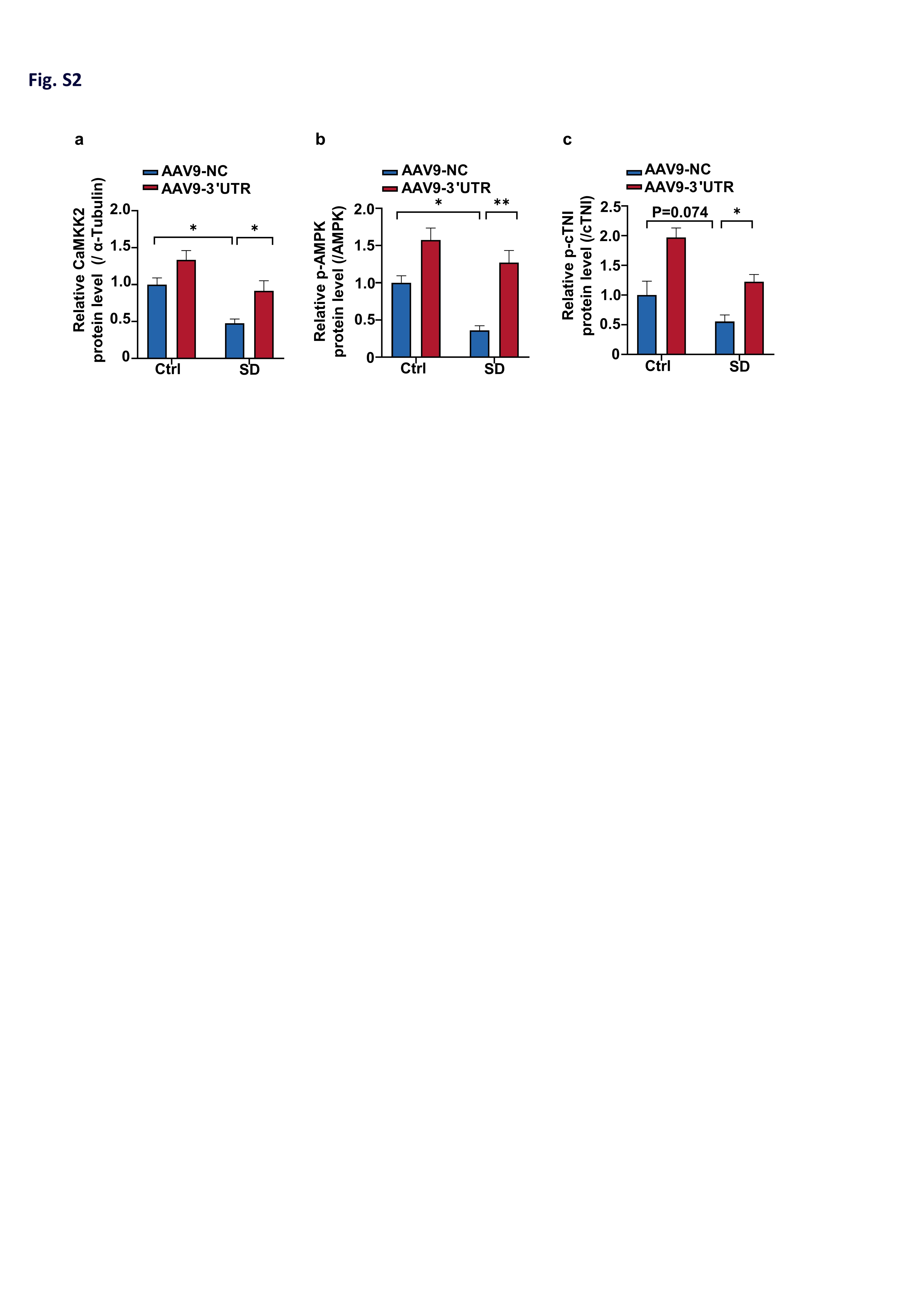

Supplement: Supplementary file 2 — Supplementary Material 2. [file 43556_2024_186_MOESM2_ESM.jpg]
